# Toluidine Blue and Chlorin-e6 Mediated Photodynamic Therapy in the Treatment of Oral Potentially Malignant Disorders: A Systematic Review

**DOI:** 10.3390/ijms26062528

**Published:** 2025-03-12

**Authors:** Anna Kruczek-Kazibudzka, Barbara Lipka, Jakub Fiegler-Rudol, Marcin Tkaczyk, Dariusz Skaba, Rafał Wiench

**Affiliations:** Department of Periodontal Diseases and Oral Mucosa Diseases, Faculty of Medical Sciences, Medical University of Silesia, 40-055 Katowice, Poland; kruczeka00@gmail.com (A.K.-K.); mtkaczyk@sum.edu.pl (M.T.); dskaba@sum.edu.pl (D.S.); rwiench@sum.edu.pl (R.W.)

**Keywords:** tolidine blue, chlorin-e6, Photolon, photodynamic therapy, PDT, OPMD, oral potentially malignant disorders, oral lichen planus, oral leukoplakia

## Abstract

Oral potentially malignant disorders (OPMDs) are conditions that carry an increased risk of malignant transformation, including oral leukoplakia and oral lichen planus. Current management approaches differ based on each condition’s unique etiology and pathophysiology, but all available treatment methods have notable limitations. This has prompted continued efforts to identify more effective therapeutic options. Photodynamic therapy (PDT) has emerged as a minimally invasive yet potent alternative for treating OPMDs. This systematic review examines the efficacy of PDT mediated by toluidine blue and chlorin-e6 (Photolon) in managing OPMDs. Following the PRISMA guidelines, eight relevant studies published between 2010 and 2024 were included. Data on the study design, protocols, light parameters, and photosensitizer characteristics were collected to evaluate treatment outcomes. The reviewed evidence suggests that toluidine-blue- and chlorin-e6-mediated PDT holds promise as a minimally invasive treatment modality for OPMDs, especially for oral lichen planus and oral leukoplakia. Studies indicate its potential as an alternative or adjunct therapy, particularly for symptomatic or refractory oral lichen planus. However, discrepancies in study designs and treatment protocols, coupled with the limited number of trials, impeded direct comparisons. Toluidine-blue- and chlorin-e6-mediated PDT shows significant potential as a therapeutic option for OPMDs. Nonetheless, further investigations—including large-scale randomized controlled trials, standardized treatment guidelines, and the exploration of additional OPMDs beyond oral lichen planus and oral leukoplakia—are necessary in order to fully establish its clinical utility and facilitate widespread adoption.

## 1. Introduction

### 1.1. Rationale

Oral potentially malignant disorders (OPMDs) cover a group of conditions affecting the oral mucosa that have an increased risk of malignant transformation into oral squamous cell carcinoma (OSCC) [1]. According to the WHO Col Collaborating Centre for Oral Cancer in the UK and updated in 2021 by Warnakulasuriya, this category encompasses a wide range of diseases including leukoplakia, erythroplakia, proliferative verrucous leukoplakia, oral lichen planus, actinic keratosis, oral submucous fibrosis, palatal lesions in reverse smokers, lupus erythematosus, dyskeratosis congenita, oral lichenoid lesions, and oral manifestations of graft-versus-host disease [2]. Graft-versus-host disease (GVHD) is a condition that occurs after allogeneic stem cell transplantation, where the donor immune cells attack the recipient’s tissues, which manifests as Lichenoid Mucositis, Xerostomia, and Mucosal Ulcerations [2,3]. Oral leukoplakia, one of the most common, affects about 3.41% of the global population, with the malignant transformation rate ranging from 3.5 to 9.8% depending on the clinical type and grade of epithelial dysplasia [3,4]. Lichen planus, on the other hand, occurs in approximately 1.01% of the global population with a 1–2% rate of malignant transformation and a risk generally described as low [5,6]. The management of OPMDs varies significantly, depending on the lesion type and underlying pathophysiology. In many cases, clinical observation and the elimination of risk factors such as smoking cessation and removing mechanical irritation are sufficient; however, oftentimes, further treatment is required [7]. In autoimmune diseases like lichen planus pharmacological intervention, priorly, a topical steroid, which is aimed at controlling the immune response, is often considered [8]. On the other hand, if the lesion presents with histologically proven dysplastic changes, which is mostly seen in oral leukoplakia or erythroplakia, the condition carries a significantly higher risk of malignant transformation and surgical intervention should be applied [9]. However, both options may have the risk of complications, for instance, scarring and functional impairment in the case of invasive treatment, or a wide range of side effects, including oral candidiasis, in the case of steroid therapy [10,11]. Additionally, the treatment may not provide a satisfactory response. This collectively underscores the need for less invasive yet effective alternatives. Photodynamic therapy (PDT) has emerged as a promising and minimally invasive option for treating both cancerous and noncancerous conditions and thus is being explored for managing various oral lesions including OPMDs, offering an effective alternative to standard treatment [12,13,14,15,16]. PTD protocol is based on the application of the photosensitizer, which accumulates in the pathological tissue and is followed by irradiation with light of the appropriate wavelength, producing reactive oxygen species (ROS) [14,15,16]. This reaction causes cytotoxic effects exclusively on the abnormal cells, while the healthy tissue remains intact [17]. The effect of PDT can be influenced by the modification of any of its elements, including the type of photosensitizer. The most frequently used photosensitizer is 5-aminolevulinic acid, a precursor to the active sensitizer protoporphyrin IX (PPIX); nonetheless, several other agents demonstrate similar properties [18]. In recent years, many compounds including methylene blue or even natural substances such as curcumin and anthocyanin have been studied, all showing promising effects as photosensitizers in PDT for OPMDs [19,20]. However, all carry limitations including potential toxicity and variability in treatment outcomes [21]. Consequently, there is a constant search for alternative photosensitizers to optimize PDT protocols and enhance therapeutic efficacy. The standard therapies for OPMDs include clinical observation with risk factor modification, topical corticosteroids for immune-mediated conditions like oral lichen planus, and surgical excision for dysplastic lesions such as leukoplakia and erythroplakia [19,20,21]. Toluidine blue and chlorin-e6 function as photosensitizers in PDT by selectively accumulating in dysplastic or inflammatory tissues [15,16,17,18,19]. Upon irradiation with an appropriate wavelength of light, they generate ROS, leading to selective cytotoxic effects on abnormal cells while preserving the surrounding healthy tissue. This mechanism promotes apoptosis, vascular damage, and immune modulation, contributing to lesion regression and symptomatic relief in OPMDs [12,13,14,15,16,17,18,19,20].

### 1.2. Objectives

In the present systematic review, we aim to evaluate the effectiveness of toluidine blue and chlorin-e6 (Photolon)-mediated PDT in the treatment of OPMDs compared to standard and alternative therapies. Our paper assesses the therapeutic potential of toluidine blue and chlorin-e6 as photosensitizers given the ongoing need to maximize treatment outcomes while ensuring minimal invasiveness. By exploring the existing evidence, we seek to determine its use in clinical practice and contribute to the advancement of PDT as a valuable option for managing oral potentially malignant disorders.

## 2. Materials and Methods

### 2.1. Focused Question

Based on the PICO framework [22], the focused question of the present systematic review was as follows: in patients with oral potentially malignant disorders (Population), is the treatment with toluidine blue or chlorin-e6 (Photolon^®^)-mediated photodynamic therapy (Intervention) effective in the management of oral potentially malignant disorders (Outcome) compared to standard therapy or alternative therapies, or was the outcome evaluated by regression/reduction in size in the follow-up period (Comparison)?

### 2.2. Search Strategy

**Figure 1:**
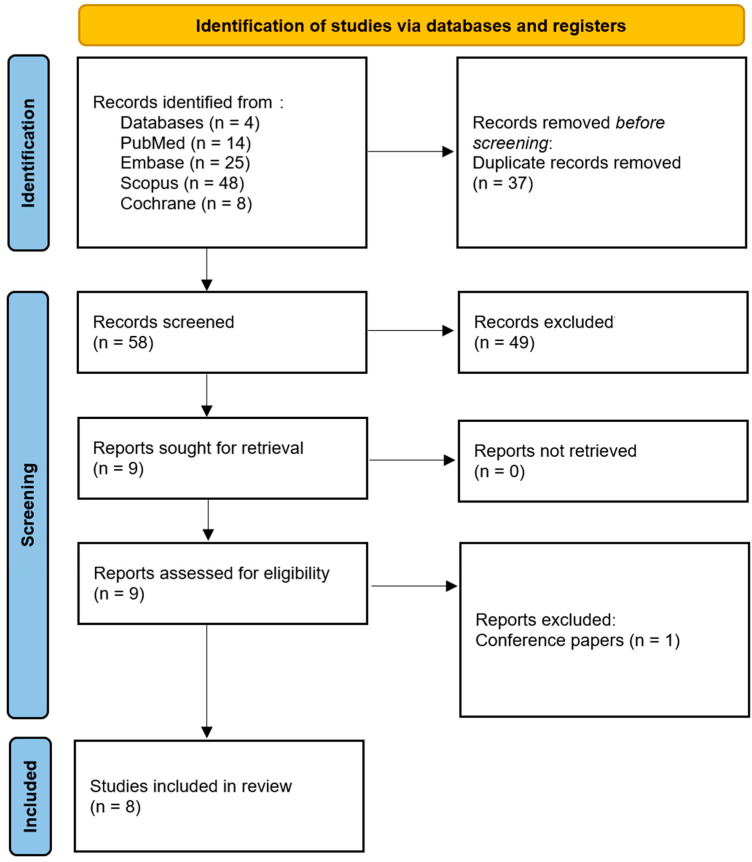
PRISMA 2020 flow diagram for new systematic reviews which included searches of databases and registers only.

This systematic review was registered with PROSPERO (ID CRD42025635999) and was conducted in accordance with Preferred Reporting Items for Systematic Reviews and Meta-Analyses (PRISMA 2020) guidelines [23]. PubMed, Embase, Scopus, and Cochrane electronic databases were searched independently by three authors, using the same search phrases consisting of keywords and Boolean operators (Table 1). To identify further relevant studies, a snowball search was performed. The eligibility criteria were established by three independent raters. In addition, only articles in English, published between 1 January 2010 and 31 December 2024, were included. After an initial search, titles and abstracts were assessed by the authors independently and checked for agreement with the involvement of a supervisor. Texts of the studies that met all the inclusion criteria were then independently read and again checked for eligibility (Table 2). Following that, selected studies were collectively discussed by the authors and any discrepancies were solved by a supervisor. Based on the discussion, with 100% concordance, included studies proceeded to data extraction (Figure 1. Prisma flow diagram).

### 2.3. Selection of Studies

Specific inclusion and exclusion criteria were formulated by the three authors independently, discussed, and then set for an agreement. During the selection process, titles, abstracts, and full-texts were first checked for eligibility by the authors independently. Then, a collective discussion was carried out, where conflicts were resolved through mediation by a third investigator. Ultimately, only the most relevant and high-quality articles were selected for further analysis.

### 2.4. Risk of Bias in Individual Studies

In the initial stage of the selection process, reviewers assessed titles, abstracts, and full-texts independently to minimize the potential risk of bias. In addition, Cohen’s kappa test was applied for every database to measure the level of inter-reviewer agreement [24]. At the final stage, collective discussion was conducted to ultimately decide on inclusion or exclusion of the selected studies. At each stage, any remaining discrepancies were resolved by a supervisor.

### 2.5. Quality Assessment

To assess the quality of the following studies, authors undertook independent screening based on criteria that were formerly collaboratively set. The evaluation criteria regarding aspects such as study design, execution, and data analysis are detailed in Table 3. The process consisted of reading each paper individually and checking for favorable factors (Table 3). If the criterion was met, the study received “1” point, and, if not, “0” points were assigned. There were 9 priorly set parameters in total, so the maximum score was 9 and the minimum was 1. The level of risk of bias was determined in accordance with the guidelines specified in the Cochrane Handbook for Systematic Reviews of Interventions and was based on points that articles received in the evaluation part [25]. Low risk was assigned for a score of 7–9 points, and moderate for 4–6 points, and 0 to 3 points indicate a high risk of bias. The risk of bias assessment for the included studies is presented in Table 3. None of the studies was excluded due to the high risk of bias. Six of eight studies were evaluated as having a low risk of bias; moreover, among these, three received the maximum score of 9. Studies that were rated moderate risk of bias were lacking a few pieces of outcome information including proper statistical analysis.

### 2.6. Data Extraction

Data extraction of the selected articles was carried out by the three authors and includes the following: citation details (first author, publication year), type of the study and country in which it was conducted, type of OPMD, type, and parameters of the light source, as well the irradiation frequency and protocol; characteristics and administration form of the photosensitizer, study design including characteristics of studied groups, evaluated parameters and follow-up period, and the outcomes of analyzed studies.

## 3. Results

### 3.1. Study Selection

The initial search yielded 95 studies. During the selection process at first, 37 duplicates were removed. After applying the eligibility criteria, 58 titles and abstracts were screened, resulting in 9 reports sought for retrieval. In the full-text assessment, one of the studies was excluded due to its type, as it was a conference paper [34]. In total, 8 studies, all published in English, between 2010 and 2024 were included in this systematic review. The PRISMA flow diagram is presented in Figure 1. The basic characteristics of the studies are covered in Table 4.

### 3.2. Data Presentation

Table 4, Table 5, Table 6 and Table 7 present details of the eight included studies that were proceeded for data extraction. Tables cover the general overview of the studies, information about the light source and photosensitizers used in PDT, and the main outcomes, which are summarized in Table 7.

### 3.3. Main Study Outcomes

The assessed studies collectively highlight that photodynamic therapy, using toluidine blue or chlorin-e6 as photosensitizers, is a promising and minimally invasive option in the management of oral potentially malignant disorders, particularly for oral leukoplakia (OL) and oral lichen planus (OLP). Jajarm et al., Lavaee et al., and Mirza et al. conducted RCTs comparing the effectiveness of toluidine-blue-sensitized PDT with topical corticosteroids for treating OLP [26,27,28]. A significant improvement in various parameters, including sign scores and pain levels, was noted in all of these studies, and PDT was therefore deemed an effective therapy for OLP. However, Jajarm et al. found that corticosteroids were more effective for pain relief [26]. Lavaee et al. highlighted the potential of TB-PDT for refractory OLP, showing a significant improvement across all measured indices [27]. In addition to PDT, Mirza et al. also evaluated the efficacy of LLLT as an alternative treatment for OLP. They found that both treatments were effective, although a greater lesion size improvement was observed in the PDT group [28]. Romano, Contaldo, et al. reported that four out of five patients showed a complete OLP lesion disappearance after TB-PDT [29]. In contrast, Muhaxheri et al. claimed that none of the five patients showed improvement, concluding that TB-PDT is not an effective option for refractory OLP [30]. Pietruska et al., Sobaniec et al., and Istomin et al. all used chlorin-e6 (Photolon) as a photosensitizer [31,32,33]. Pietruska et al. and Sobaniec et al. followed a similar study design in which all patients were treated with C-e6-PDT, and efficacy was evaluated according to the authors’ own method, which involved dividing the lesions into five size groups [31,32]. Pietruska et al. found that C-e6-PDT significantly reduced the oral leukoplakia lesion size, with a 53.8% average reduction and complete regression in 12 of 44 sites [31]. Sobaniec et al. demonstrated that C-e6-PDT is an efficacious and minimally invasive treatment for OLP, with the best outcomes observed in patients over 75 years old [32]. Regardless of lesion type, both studies found the treatment to be particularly effective for lesions on the buccal and lip mucosa [31,32]. Istomin et al. investigated photon-mediated PDT, where the photosensitizer is administered intravenously, and found it to be a highly effective therapy for oral leukoplakia, achieving a 95% rate of complete regression [33]. The study also proved that, when performed under local anesthesia, photolon-mediated PDT is a safe and well-tolerated option. Additionally, the temporary necrosis that occurred directly after irradiation resulted in full epithelialization within 3 to 6 weeks.

## 4. Discussion

### 4.1. Results in the Context of Other Evidence

The results of this systematic review proposed that, while toluidine blue or chlorin-e6 (Photolon^®^)-mediated photodynamic therapy (PDT) is an effective treatment for oral potentially malignant disorders (OPMDs), Regarding this postulation, the findings of the reviewed studies provide substantial evidence supporting the efficacy of toluidine-blue-or chlorin-e6-mediated PDT in treating OPMDs, particularly oral lichen planus and oral leukoplakia, highlighting its potential as an alternative or complementary therapy [26,27,28,29,30,31,32,33]. However, differences in the study design and treatment protocols present a complex landscape for a direct comparison between interventions—standard (topical glucocorticoids) or alternative (LLLT) therapies. RCTs conducted by Jajarm et al., Lavaee et al., and Mirza et al. compared toluidine-blue-sensitized PDT to topical corticosteroids in treating OLP [26,27,28]. All of these studies deemed PDT a valuable alternative or adjunct to conventional steroid therapy, reporting significant improvements in lesion sign scores and pain relief. However, both Jajarm et al. and Mirza et al. found corticosteroids to be superior for pain reduction [26,28]. These findings align with a systematic review by Gulzar et al., which compared PDT and corticosteroid therapy in the management of OLP, concluding that PDT is a promising option for treating symptomatic OLP lesions, as it provides significant symptom reduction and improves functionality [35]. Mirza et al. extended the scope of comparison by including low-level laser therapy (LLLT), which, like PDT, showed significant efficacy [28]. However, a stronger improvement in lesion size was noted in the PDT group. Several other authors who also assessed the effectiveness of LLLT in OLP [36,37,38,39] confirmed that LLLT significantly improves sign scores and pain levels. Jajarm et al., who conducted a systematic review on the effectiveness of LLLT and PDT therapy in OLP, also compared both treatments to standard steroid therapy, finding significant differences in LLLT effects on severity. Nevertheless, there is a lack of direct comparison between blue light treatment and low-level laser therapy [40]. Lavaee et al. highlighted the potential of TB-PDT as an alternative therapy for refractory OLP, a finding that was also demonstrated in a case series of 10 patients, where Rakesh et al. used 5-aminolevulinic acid as a sensitizer and reported remarkable improvement in several scores for refractory OLP [27,41]. In contrast, Muhaxheri et al. reported that none of the five patients with refractory OLP showed improvement. Nonetheless, this evidence may be limited due to the small sample size [30]. In several studies, photochemotherapy proved to be so effective that it led to a complete regression of the lesion. Peralta-Mamani et al., who studied the clinical efficacy of PDT in oral premalignant lesions, reported a great prevalence of total remission, which yielded 44% for oral leukoplakia, 69.9% for actinic cheilitis, 92.1% for oral erythroleukoplakia, and 98.5% for oral verrucous hyperplasia [42]. Similar findings were observed by Romano, Contaldo et al., Pietruska et al., and Sobaniec et al. [29,31,32]. Istomin et al., in whose study the photosensitizer was administered intravenously, even claimed a 95% regression rate [33]. This suggests that better results might be obtained when the photosensitizer is administered intravenously. Jerjes et al., who also injected the photosensitizer Temoporfin (mTHPC), achieved a remarkable 81% complete response in patients with erythroplakia or leukoplakia [43]. However, a direct conclusion cannot be drawn due to the insufficient number of studies utilizing intravenous administration. Additionally, the application of PDT shows variability depending on the lesion location. Both Pietruska et al. and Sobaniec et al. observed that PDT was more effective in treating lesions on the buccal and lip mucosa [31,32]. Wang et al. also noted differences in treatment outcomes across various sites, showing that the buccal mucosa was a protective factor against the recurrence of oral leukoplakia [44]. Several of the reviewed studies also demonstrated that TB- and C-e6-mediated PDT is particularly valuable for the atrophic, erosive, and ulcerative forms of oral lichen planus [26,27,28]. This is further supported by the systematic reviews conducted by Akram et al. and Al-Maweri et al., who both evaluated PDT for symptomatic oral lichen planus and highlighted its great potential [45,46]. In the reviewed studies, toluidine-blue- and chlorin e6-mediated PDT was exclusively applied to oral lichen planus and oral leukoplakia [26,27,28,29,30,31,32,33]. However, the broader literature extends the scope of OPMDs targeted by photochemotherapy to include oral erythroplakia, oral erythroleukoplakia, oral verrucous hyperplasia, and actinic cheilitis. These conditions, however, have been treated using other photosensitizers, mainly 5-aminolevulinic acid [42,47,48]. Therefore, to fully assess the potential of toluidine blue and chlorin-e6 as photosensitizers, further studies are needed to explore their use in a wider range of oral premalignant lesions. Considering safety, PDT has proven to be a safe and minimally invasive treatment, even when the photosensitizer is administered intravenously [26,27,28,29,30,31,32,33]. Furthermore, Istomin et al. demonstrated that, under local anesthesia, photodynamic therapy is well-tolerated and that the temporary necrosis observed immediately after the irradiation leads to full epithelialization within 3 to 6 weeks [33]. This conclusion is consistently supported across the literature, with multiple authors affirming PDT as a safe treatment with minimal side effects [42,48,49]. Overall, the reviewed studies collectively underscore the potential of toluidine-blue- and chlorin-e6-mediated photochemotherapy as an effective, safe, and minimally invasive therapy for managing oral premalignant lesions, which may serve as an alternative or complementary treatment, even in refractory cases [26,27,28]. Variations in the light source parameters, such as wavelength, power density, and irradiation time, alongside the method of photosensitizer administration (topical vs. intravenous), significantly influenced treatment outcomes, with intravenous administration demonstrating higher lesion regression rates and enhanced efficacy, while lesion location also impacted treatment success, particularly favoring buccal and lip mucosa sites [23,24,25,26,27,28,29,30,31,32,33,34,35,36,37,38,39,40]. Despite their promising therapeutic effects, both toluidine blue and chlorin-e6 have certain limitations. Toluidine blue exhibits variability in tissue penetration, limiting its efficacy in deeper lesions. Chlorin-e6, while demonstrating enhanced selectivity and penetration, may require prolonged incubation times and specific light parameters for optimal activation [26,27,28,29,30,31,32,33]. Additionally, the standardization of PDT protocols using these agents remains inconsistent across studies, making direct comparisons challenging. Further, the limited long-term follow-up data restrict our understanding of recurrence rates and sustained therapeutic effects [26,27,28,29,30,31,32,33].

### 4.2. Limitations of the Evidence

The studies discussed in this systematic review have several limitations, which could impact the generalizability of the findings. The major one lies in the diversity of study designs and treatment protocols. While there were three RCTs, with mostly coherent designs, for the rest of the studies, models significantly varied, which enabled direct comparisons. Moreover, not all of the studies included a comparison group, making it difficult to isolate the proper effects of the PDT. There were also different types of outcome assessment, and, in some papers, even their own method was applied, which could introduce a risk of bias. Regarding the study protocol, the parameters of the light source as well the type of photosensitizer administration differed across studies. Another key limitation occurred in the small group sizes. Most studies had fewer than 50 participants, limiting the statistical power of the conclusions. Besides that, two studies lacked some outcome data, which might have affected the overall results. The last limitation lies in the limited duration of the follow-up, which, across the studies, did not exceed 6 months, and does not allow for the assessment of long-term outcomes.

### 4.3. Limitations of the Review Process

In the present review, substantial heterogeneity was observed among the included studies, with notable differences in study designs, treatment protocols, sample sizes, follow-up periods, and outcome assessment methods. Along with the limited number of studies assessing the effectiveness of TB- and C-e6-mediated PDT for OPMDs, this lack of homogeneity prevented the authors from drawing strong conclusions. Moreover, the variability in PDT parameters across studies hindered the application of the GRADE tool, making it challenging to determine the most effective PDT protocol. Additionally, the exclusion of non-English literature may have narrowed the scope of the review. Overall, the discussed issues may have introduced a risk of bias and restrained direct comparisons, thereby limiting the ability to perform a meta-analysis. Consequently, these limitations led to a narrative synthesis of the findings. Furthermore, the findings collectively underscore the need for future research involving larger-scaled, well-designed randomized controlled trials to expand the scope of analysis, establish standardized protocols, and enable quantitative and systematic comparisons.

### 4.4. Implications for Practice, Policy, and Future Research

Both toluidine blue (TB)- and chlorin-e6 (C-e6)-mediated PDT presented promising results in managing oral potentially malignant disorders (OPMDs), particularly oral lichen planus (OLP) and oral leukoplakia (OL). These therapies demonstrated effectiveness along with minimal invasiveness and, therefore, should be considered as an alternative or a complementary treatment. Particularly promising results were observed in cases of refractory or symptomatic lichen planus, highlighting PDT as a valuable approach for these instances. However, to promote further implementation into clinical practice, there is a need to expand research to establish standardized protocols and develop official treatment guidelines. Policymakers and funding agencies should support large-scale, multicenter randomized controlled trials to optimize PDT protocols across different populations. An emphasis should also be put on coherent study designs which would enhance the consistency and enable direct comparisons and the maximization of the effectiveness of PDT. Furthermore, research is needed in order to explore its cost-effectiveness and patient accessibility. Future studies should focus on comparing toluidine-blue- and chlorin-e6-mediated photodynamic therapy to both standard and alternative therapies. There is also a need to extend the scope to other oral premalignant lesions besides oral leukoplakia and lichen planus to fully assess the potential of this therapy for treating OMPDs. Researchers should also focus on the optimization of the protocol including safety and the laser parameters, and investigate PDT in combination with other treatments. Moreover, patient-specific aspects such as the variability of the location should be further explored. The research should also include long-term efficacy as well as the impact on preventing malignant transformation. Future research should focus on optimizing PDT protocols with toluidine blue and chlorin-e6 to enhance treatment efficacy and consistency. Investigations into combination therapies—such as PDT with immunomodulators or low-level laser therapy—could improve outcomes, particularly for refractory cases. Additionally, exploring novel delivery systems, such as nanoparticle-based formulations, may enhance photosensitizer penetration and bioavailability. Long-term clinical trials are necessary in order to assess sustained efficacy, potential recurrence rates, and the role of PDT in reducing malignant transformation risks in OPMDs.

To facilitate the integration of toluidine-blue- and chlorin-e6-mediated PDT into routine clinical practice, regulatory bodies and professional organizations should establish standardized treatment protocols based on robust clinical evidence. Policymakers should support the development of evidence-based guidelines that define optimal light parameters, dosage, and administration protocols to enhance treatment reproducibility. Additionally, insurance and healthcare systems should evaluate the cost-effectiveness of PDT to promote broader accessibility. Multicenter randomized controlled trials should be prioritized through funding initiatives to validate PDT as a frontline or adjunctive therapy for OPMDs, ensuring its adoption into national and international clinical guidelines.

## 5. Conclusions

This systematic review presents the potential of toluidine-blue- and chlorin-e6-mediated photodynamic therapy as a promising, effective, and minimally invasive treatment for oral potentially malignant disorders, particularly oral lichen planus and oral leucoplakia. PDT has proven to be a valuable alternative or complementary treatment option, especially for symptomatic and refractory cases of oral lichen planus. Significant improvements in various parameters, including sign scores and pain levels, have been observed across the studies. The research has also proven toluidine-blue- and chlorin-e6-mediated PDT to be a safe therapy. These findings collectively highlight the potential of PDT and advocate for its further implementation into clinical practice. However, differences in study designs and treatment protocols which hindered direct comparisons, along with a limited number of studies including an insufficient number of large-scale randomized controlled trials, may have impacted the generalizability of the findings, consequently limiting their applicability. The limitations underscore the need for further research to prioritize large-scale, multicenter randomized controlled trials with coherent and consistent study designs. This would enable the optimization of PDT protocols, maximize effectiveness, and establish standardized guidelines. Furthermore, to fully assess the potential of toluidine-blue- and chlorin-e6-mediated PDT for OPMDs, research should expand its scope to include other types of premalignant oral conditions besides oral leucoplakia and oral lichen planus. Addressing these critical gaps could help further implementation and broaden the use of PDT in clinical practice.

## Figures and Tables

**Table 1:** Search syntax used in the study.

Source	Search Term	Number of Results
PubMed	((“Toluidine Blue” OR “Chlorin-e6” OR “Chlorine-e6” OR “dimethyl sulfoxide” OR “Photolon”) AND ((“Photodynamic Therapy”) OR “PDT” OR “Photochemotherapy” OR “aPDT” OR “Antimicrobial Photodynamic Therapy”) AND ((“Lichen Planus, Oral”) OR “Oral Lichen Planus” OR “ORL” OR “Leukoplakia, Oral” OR “Oral Leukoplakia” OR “Erythroplakia” OR “Erythroleukoplakia” OR “Erythroplasia” OR “Proliferative verrucous leukoplakia” OR “Oral verrucous leukoplakia” OR “Oral Submucous Fibrosis” OR “Smokers palate” OR “Reverse smoking” OR “Oral lupus erythematosus” OR “Discoid Lupus Erythematosus” OR “Actinic Cheilitis” OR “Actinic Keratosis” OR “dyskeratosis congenita” OR “Oral Graft versus Host Disease” OR “OGVHD” OR “Oral GVHD” OR “Oral Lichenoid Lesions” OR ”Oral potentially malignant disorders” OR “OPMD” OR “Oral Premalignant Lesions” OR “Oral potentially malignant lesions” OR “Oral precancerous lesions” OR “PMD”))	14
Embase	(‘Toluidine Blue’ OR ‘Chlorin-e6′ OR ‘Chlorine-e6′ OR ‘dimethyl sulfoxide’ OR ‘Photolon’ AND ((‘Photodynamic Therapy’ OR ‘PDT’ OR ‘photochemotherapy’ OR ‘aPDT’ OR ‘Antimicrobial Photodynamic Therapy’):ti,ab) AND ((‘Lichen Planus, Oral’ OR ‘Oral Lichen Planus’ OR ‘ORL’ OR ‘Leukoplakia, Oral’ OR ‘Oral Leukoplakia’ OR ‘Erythroplakia’ OR ‘Erythroleukoplakia’ OR ‘Erythroplasia’ OR ‘Proliferative verrucous leukoplakia’ OR ‘Oral verrucous leukoplakia’ OR ‘Oral Submucous Fibrosis’ OR ‘Smokers palate’ OR ‘Reverse smoking’ OR ‘Oral lupus erythematosus’ OR ‘Discoid Lupus Erythematosus’ OR ‘Actinic Cheilitis’ OR ‘Actinic Keratosis’ OR ‘dyskeratosis congenita’ OR ‘Oral Graft versus Host Disease’ OR ‘OGVHD’ OR ‘Oral GVHD’ OR ‘Oral Lichenoid Lesions’ OR ‘Oral potentially malignant disorders’ OR ‘OPMD’ OR ‘Oral Premalignant Lesions’ OR ‘Oral potentially malignant lesions’ OR ‘Oral precancerous lesions’ OR ‘PMD’)):ti,ab)	25
Scopus	TITLE-ABS-KEY ((“Toluidine Blue” OR “Chlorin-e6” OR “Chlorine-e6” OR “dimethyl sulfoxide” OR “Photolon”) AND (“Photodynamic Therapy” OR “PDT” OR “Photochemotherapy” OR “aPDT” OR “acrobial Photodynamic Therapy”) AND (“Lichen Planus, Oral” OR “Oral Lichen Planus” OR “ORL” OR “Leukoplakia, Oral” OR “Oral Leukoplakia” OR “Erythroplakia” OR “Erythroleukoplakia” OR “Erythroplasia” OR “Proliferative verrucous leukoplakia” OR “Oral verrucous leukoplakia” OR “Oral Submucous Fibrosis” OR “Smokers palate” OR “Reverse smoking” OR “Oral lupus erythematosus” OR “Discoid Lupus Erythematosus” OR “Actinic Cheilitis” OR “Actinic Keratosis” OR “dyskeratosis congenita” OR “Oral Graft versus Host Disease” OR “OGVHD” OR “Oral GVHD” OR “Oral Lichenoid Lesions” OR “Oral potentially malignant disorders” OR “OPMD” OR “Oral Premalignant Lesions” OR “Oral potentially malignant lesions” OR “Oral precancerous lesions” OR “PMD”))	48
Cochrane	((“toluidine blue” OR chlorine-e6 OR chlorin-e6 OR “dimethyl-sulfoxide” OR photolon):ti,ab,kw) and ((“photodynamic therapy” OR “PDT” OR “photochemotherapy” OR “antimicrobial photodynamic therapy” OR “aPDT”):ti,ab,kw) AND ((“oral lichen planus” OR “oral leukoplakia” OR “oral erythroplakia” OR “oral erythroplasia” OR “erythroleukoplakia” OR “proliferative verrucous leukoplakia” OR “oral verrucous leukoplakia” OR “oral submucous fibrosis” OR “smokers palate” OR “reverse smoking” OR “oral lupus erythematosus” OR “discoid lupus erythematosus” OR “actinic cheilitis” OR “actinic keratosis” OR “dyskeratosis congenita” OR “oral graft versus host disease” OR “OGVHD” OR “Oral GVHD” OR “oral lichenoid lesion” OR “oral potentially malignant disorders” OR “OPMD” OR “oral premalignant lesions” OR “oral potentially malignant lesions” OR “oral precancerous lesions” OR “PMD”):ti,ab,kw)	8

**Table 2:** Selection criteria for papers included in the systematic review.

Inclusion Criteria	Exclusion Criteria
Adequate OMPD diagnosis clinically or histopathologically confirmed; Randomized trials/randomized controlled trials; Studies where toluidine blue or chlorin-e6 (Photolon) was used as the main photosensitizer in photodynamic therapy for OPMD; Studies that compare the efficacy of toluidine blue or chlorin-e6 (Photolon) sensitized PDT for OPMD with standard treatments/alternative treatments; Longitudinal studies/studies with follow-up monitoring the outcome of toluidine blue or chlorin-e6 (Photolon) sensitized photodynamic therapy for OPMD.	Case reports, case series, letters to the editor, narrative or systematic reviews and meta-analyses, unpublished articles, and conference papers; Publications in languages other than English; Non-peer-reviewed studies; Studies assessing lesions with insufficient evidence to be included in the category of OMPD (established in 2007 by the World Health Organization (WHO) Collaborating Centre for Oral Cancer in the UK and updated in 2021 by Warnakulasuriya [2]); Studies assessing cancerous lesions and general medical applications unrelated to OPMD; “Grey literature”.

PDT—photodynamic therapy; OPMD—oral potentially malignant disorder.

**Table 3:** The risk of bias across studies and detailed evaluation criteria.

	Study							
Evaluation Criteria	Jajarm et al. (2015) [26]	Lavaee et al. (2019) [27]	Mirza et al. (2018) [28]	Romano, Contaldo et al. (2019) [29]	Muhaxheri et al. (2017) [30]	Pietruska et al. (2014) [31]	Sobaniec et al. (2013) [32]	Istomin et al. (2016) [33]
1. Concentration of the photosensitizer mentioned/provided by stating the photosensitizer trade name	1	1	1	0	0	1	1	1
2. The form of photosensitizer administration as well as the incubation time mentioned	1	1	1	1	1	1	1	1
3. All significant parameters of the light source such as type of laser, wavelength, energy fluence, and power density mentioned	1	1	1	0	1	0	0	1
4. Information about the frequency of the irradiation as well as the number of sessions included	1	1	1	1	1	1	1	0
5. Adequate OPMD diagnosis histopathologically confirmed	1	1	1	1	0	1	1	1
6. Site of the OMPD detailed	1	1	1	1	1	1	1	1
7. Inclusion/exclusion criteria defined	1	1	1	1	0	1	0	0
8. No missing outcome data	1	1	1	0	0	1	1	1
9. Statistical analysis implied	1	1	1	0	0	1	1	1
Total score	9	9	9	5	4	8	7	7
Risk of Bias	Low	Low	Low	Moderate	Moderate	Low	Low	Low

OPMD—oral potentially malignant disorder.

**Table 4:** General characteristics of the studies.

First Author(s) and Reference	Year	Country	Model of the Study	Photosensitizer	Type of OPMD
Jajarm et al. [26]	2015	Iran	in vivo	toluidine blue	oral lichen planus
Lavaee et al. [27]	2019	Iran	in vivo	toluidine blue	oral lichen planus
Mirza et al. [28]	2018	Pakistan	in vivo	toluidine blue	oral lichen planus
Romano, Contaldo et al. [29]	2019	Italy	in vivo	toluidine blue	oral lichen planus
Muhaxheri et al. [30]	2017	Croatia	in vivo	toluidine blue	oral lichen planus
Pietruska et al. [31]	2014	Poland	in vivo	chlorin-e6/Photolon^®^	oral leukoplakia
Sobaniec et al. [32]	2013	Poland	in vivo	chlorin-e6/Photolon^®^	oral lichen planus
Istomin et al. [33]	2016	Belarus	in vivo	chlorin-e6/photolon	oral leukoplakia

**Table 5:** Characteristics of the light source used in PDT.

Study and Reference	Type of the Laser	Wavelength [nm]	Energy Fluence [J/cm^2^]	Power Density [mW/cm^2^] /Power [mW]	Irradiation Time [min]	Frequency	Number of Sessions	Interval Time	Spot Surface [cm^2^]/Diameter [cm]/Tip
Jajarm et al. [26]	GaAlAs diode laser (Mustang 2000, Russia)	630	1.5	10 mW/cm^2^	2.5	continuous	-	2 × weekly for 1 month	1 cm^2^
Lavaee et al. [27]	InGaAlP diode laser (Azor-2k, Russia)	660	19.23	25 mW	10	spot	3	1 week	0.78 cm^2^
Mirza et al. [28]	GaAlAs diode laser	630	1.5	10 mW/cm^2^	2.5	continuous	-	2 × weekly for 1 month	1 cm^2^
Romano, Contaldo et al. [29]	FotoSan^®^	630	-	-	2.5	-	2–5	14 days minimum	-
Muhaxheri et al. [30]	GaAlAs diode laser	685	2.00	30 mW	-	continuous	6	2–3 days (irradiation on days: 1, 3, 5, 8, 10, 12)	1 cm^2^
Pietruska et al. [31]	semiconductor laser (Haemato, Poland)	660	90	300 mW	-	-	10	2 weeks	diffuser tip
Sobaniec et al. [32]	semiconductor laser (Haemato Poland)	660	90	300 mW	-	-	10	2 weeks	diffuser tip
Istomin et al. [33]	semiconductor laser UPL PDT (LEMT, Belarus)	660	25–100	0.07–0.32 W/cm^2^	2.5–13	-	1–3	-	1 cm

**Table 6:** Characteristics of photosensitizer used in PDT.

Study and Reference	Photosensitizer	Administration	Concentration/Dose	Application Protocol	Incubation Time [min]	Trade Name of the PS
Jajarm et al. [26]	TB	topical	1 mg/mL	micropipette (50 μL in total)	10	-
Lavaee et al. [27]	TB	topical	1 mg/mL	sterile swab	10	-
Mirza et al. [28]	TB	topical	1 mg/mL	micropipette (50 μL in total)	10	-
Romano, Contaldo et al. [29]	TB	topical	-	2% AA (1 min) → drying with gauze → TB → 1% AA (1 min)	-	-
Muhaxheri et al. [30]	TB	topical	-	cotton stick	10	-
Pietruska et al. [31]	C-e6	topical	20% C-e6, 10% DS	occlusive dressing on a dried mucosa: C-e6 on a nonwoven fabric, covered with a polyethylene sheet, and a sterile gauze	60	Photolon^®^ (Haemato, Poland)
Sobaniec et al. [32]	C-e6	topical	20% C-e6, 10% DS	occlusive dressing on a dried mucosa: C-e6 on a nonwoven fabric, covered with a polyethylene sheet, and a sterile gauze	60	Photolon^®^ (Haemato, Poland)
Istomin et al. [33]	C-e6	i.v.	1.7–2.5 mg/kg	intravenously in the darkened room	150–180	photolon

PS—photosensitizer; TB—toluidine blue; C-e6—chlorin-e6; AA—acetic acid; DS—dimethyl sulfoxide.

**Table 7:** Main outcomes and details from each study.

Study and Reference	Patients	M:F	Age/Mean Age + SD [yr]	Site of the Lesions	Type of the Lesions (Number of Affected Patients)	Study Design/Treatment Protocol	Results
Jajarm et al. [26]	25 randomly allocated: 11 experimental group, 14 control group	8:17 3:8 5:9	-	T or BM	OLP; atrophic-erosive	RCT; comparing the effect of TB-PDT with topical CS in the treatment of OLP; experimental group: TB-PDT for 1 month; control group: topical corticosteroids (dexamethasone, 0.5 mg/5 mL water, in mouthwash), four times a day for 1 month. Sign scores were assessed using the Thongprasom sign scoring. Improvement of the lesions was measured by applying efficacy indices (EI). Evaluation of experienced pain was assessed using the 1–10 VAS scale. During the treatment, effects were evaluated weekly, and after completion of the treatment, a 2-, 3-, and 4-week follow-up was included.	The study demonstrated that TB-PDT was effective in the management of OLP. Sign scores of changes significantly decreased after the treatment in both groups: experimental (*p* = 0.021), control (*p* = 0.002); however, there was no significant difference (*p*= 0.72) between the groups. The intensity of lesions significantly reduced after treatment in both groups, experimental (*p* = 0.005), and control (*p*= 0.001), and a significant difference between the groups was observed (*p* = 0.001). The mean amount of improvement in pain was significantly higher in the control group (*p* < 0.001) (α = 0.05). In the follow-up statistically significant difference was observed (*p* = 0.042), as a relapse did not show in 100% of patients in the control group, compared to 72.7% of patients in the experimental group.
Lavaee et al. [27]	11 (22 sides) randomly, double- blind allocated: intervention side (11), control side (11)	2:9	-	bilateral OM	OLP; symptomatic atrophic/ erosive/ulcerative	BRCT; comparing the effect of TB-PDT with topical CS in the treatment of OLP; intervention side: all patients underwent TB-PDT in 3 sessions in one-week intervals—session 0, 1, and 2, respectively; alongside sham laser irradiation for the control side. On the third-week follow-up (session 3), an effect of the TB-PDT was evaluated and topical CS (triamcinolone acetonide 0.1%) was prescribed for 4 weeks in all patients. On the seventh week, follow-up (session 4) outcome assessment was held. Sign scores were assessed using the Thongprasom sign scoring (TH); the symptoms (pain) were evaluated with the 1–10 VAS scale. Moreover, clinical severity index (SI) and efficacy index (EI) were determined.	The major conclusion was that TB-PDT can be used as an alternative therapy besides standard methods, and can be considered as a new approach for refractory OLP. In the final assessment, the results of 8 patients were evaluated (3 patients were excluded). For the intervention side, statistically significant improvement between sessions 0 and 4 in all scores (VAS, TH, SI, and EI) was observed (*p* value < 0.05). Except for the TH score in the control side (*p* value = 0.056), differences between the changes in all indices were statistically significant between session 0 and 4 (*p* value < 0.05).
Mirza et al. [28]	45 randomly allocated: 15-PDT group, 15-LLLT group, 15-GS group	8:37 3:12 1:14 4:11	52.6 ± 11.4 50.8 ± 14.7 49.2 ± 10.6	T or BM	OLP; atrophic-erosive	RCT; comparing the effects of TB-PDT, LLLT, or topical GS in the treatment of OLP; group 1: TB-PDT in two sessions, two times weekly for 1 month; group 2: LLLT in maximum of 10 sessions, two times weekly (three-day intervals), group 3: topical CS (dexamethasone, 0.5 mg/5 mL water) four times a day for 1 month. Sign scores were evaluated using the Thongprasom sign scoring; moreover, an efficacy index (EI) was determined. 1–10 VAS scale was applied to evaluate the symptoms (pain).	The study proved that TB-PDT and LLLT were effective in the management of erosive-atrophic OLP. A significant difference in sign score change before and after the treatment in TB-PDT group (*p* = 0.03), LLLT group (*p* = 0.04), and CS group (*p* = 0.02) was observed. Moreover, statistically significant difference was noted between TB-PDT group (*p* = 0.001) and LLLT group (*p* = 0.001) before and after the treatment. This tendency did not show in CS group. Mean improvement in pain was significantly higher in the GS group (*p* < 0.001). The EI improvement in the TB-PDT group was significantly greater compared to LLLT and GS groups.
Romano, Contaldo et al. [29]	5	2:3	62–71	upper adherent G	OLP; multifocal, homogenous	Study assessed the feasibility of TB-PDT in the treatment of OLP. All patients were treated with TB-PDT. The efficacy was assessed using the authors’ own method, which included measuring the lesion (in mm) on each session.	Outcomes of the study indicate that TB-PDT is a valuable treatment option for OLP. Complete response (total disappearance of clinically visible lesions) was noted in four patients. One patient presented with a partial response—54% reduction in size (T0 = 22 mm; Tf = 12 mm).
Muhaxheri et al. [30]	5	1:4	50–80	AR (2); G (1); BM + AR (1); T (1)	OLP; refractory	The study aimed to evaluate the efficacy of TB-PDT for the treatment of refractory OPL. All patients were treated with TB-mediated PDT.	The study found that PDT with toluidine blue was not effective in five patients with refractory OLP.
Pietruska et al. [31]	23 (44 lesions)	7:16	21–79	BM + L (38); G + T (6)	OL; homogenous flat	The study evaluated the efficacy of C-e6-PDT in the treatment of OL. All patients were treated with C-e6-PDT. Efficacy was assessed by measuring the lesion (in mm) at 1, 2, 5, and 10 sessions. To evaluate the efficacy, the authors’ own method was applied, in which lesions were divided into 5 groups. Additionally, gender- and age-related data, and smoking as a factor were analyzed.	Researchers proved that C-e6-PDT results in a significant size reduction in OL lesions. A size reduction was noted in 34 of 44 sites; 12 sites presented with a complete regression. The mean size reduction of OL lesions was statistically significant (by 53.8%). In all groups (M, F, smokers, and nonsmokers) a statistically significant reduction in the lesion size on the buccal and lip mucosa was observed. The differences between age groups were insignificant.
Sobaniec et al. [32]	23 (48 lesions)	6:17	31–82	BM + L (40); G + T (8)	OLP	The study assessed the efficacy of C-e6-PDT in the treatment of OLP. All patients were treated with C-e6-PDT. Efficacy was assessed by measuring the lesion (in mm) at 1, 2, 5, and 10 sessions. The efficacy was evaluated according to the authors’ own method, in which lesions were classified into 5 size groups. Additionally, gender- and age-related data, and smoking as a factor were analyzed.	C-e6-PDT turned out to be an efficient and non-invasive treatment for OLP. The size reduction was noted in 39 of 48 sites, including 14 with a complete regression. The mean size reduction in OLP lesions was statistically significant (by 55%). The greatest, statistically significant effects were noted for the lesions on the buccal and lip mucosa (reduction by 57.6%) for all the groups (M, F, smokers, nonsmokers). C-e6-PDT was statistically significantly less effective for the lesions on the gingiva and tongue (30.0% reduction). Regarding age, the best effects were observed for the lesions on the buccal and lip mucosa in patients over 75 years (by 66.9%).
Istomin et al. [33]	40 (109 lesions)	7:33	55 ± 14	BM (17); T (15); G (5); OF (3)	OL; flat (38), verrucous (2)	The study evaluated the tolerability, safety, and immediate results of photon-mediated PDT for the treatment of OL. All patients underwent photolon-mediated PDT, which was administered intravenously; 15–20 min before the treatment, local anesthesia was carried out. Additionally, fluorescence spectrophotometry was used to plan, monitor, and optimize the PDT. After completion of the PDT, a 7-day, and 1-, 3-, and 6-month follow-up was included. The evaluation of immediate results was carried out according to the WHO criteria: complete regression, partial regression (by 50% in size or more), and no effect (less than 50% or no reduction in size). The final evaluation was held 1–2 months after completion of the PDT.	Researchers proved that PDT may be considered an effective and well-tolerated option for the treatment of circumscribed or widespread OL. On the 1–2-month follow-up, 38 of 40 patients (95%) presented with a complete regression. In 2 cases, all with verrucous type, partial regression was observed. There were no complications regarding the photolon administration. During the PDT, patients reported local pain sensation, which was well managed with local anaesthesia (ketorolac, i.m. or 2% lidocaine, topically). On days 2–6 after the completion of PDT, hemorrhagic necrosis was observed in the area of treated lesions. All the affected spots presented with full epithelialization after 3–6 weeks.

RCT—randomized controlled trial; BRTC—blinded randomized controlled trial M—man; F—female; PDT—photodynamic therapy; TB-PDT—toluidine-blue-mediated photodynamic therapy, C-e6-PDT—chlorin-e6-mediated photodynamic therapy, CS—corticosteroid; LLLT—low-level laser therapy; OLP—oral lichen planus; OL—oral leukoplakia; VAS—visual analogue scale; TH—Thongprasom sign scoring; PS -photosensitizer; yr—years; T—tongue; BM—buccal mucosa; OM—oral mucosa; G—gingiva; OF—oral floor, AR—alveolar ridge; L—lips.
